# Principles for urban nature-based solutions

**DOI:** 10.1007/s13280-021-01685-w

**Published:** 2022-01-17

**Authors:** Nadja Kabisch, Niki Frantzeskaki, Rieke Hansen

**Affiliations:** 1 Department of Geography, Humboldt-Universität zu Berlin, Unter den Linden 6, 10099 Berlin, Germany; 2 Department Urban and Environmental Sociology, Helmholtz Centre for Environmental Research – UFZ Leipzig, Permoserstrasse 12, 04318 Leipzig, Germany; 3 Centre for Urban Transitions, School of Arts, Social Sciences and Humanities, Faculty of Health, Arts and Design, Swinburne University of Technology, Melbourne, Australia; 4 Department of Open Space Development, Hochschule Geisenheim University, Von-Lade-Str. 1, 65366 Geisenheim, Germany

**Keywords:** Biodiversity, Cities, Climate change, Environmental justice, Governance, Sustainability

## Abstract

Nature-based solutions (NBS) were introduced as integrated, multifunctional and multi-beneficial solutions to a wide array of socio-ecological challenges. Although principles for a common understanding and implementation of NBS were already developed on a landscape scale, specific principles are needed with regard to an application in urban areas. Urban areas come with particular challenges including (i) spatial conflicts with urban system nestedness, (ii) specific urban biodiversity, fragmentation and altered environments, (iii) value plurality, multi-actor interdependencies and environmental injustices, (iv) path-dependencies with cultural and planning legacies and (v) a potential misconception of cities as being artificial landscapes disconnected from nature. Given these challenges, in this perspective paper, we build upon and integrate knowledge from the most recent academic work on NBS in urban areas and introduce five distinct, integrated principles for urban NBS design, planning and implementation. Our five principles should help to transcend governance gaps and advance the scientific discourse of urban NBS towards a more effective and sustainable urban development. To contribute to resilient urban futures, the design, planning, policy and governance of NBS should (1) consider the need for a systemic understanding, (2) contribute to benefiting people and biodiversity, (3) contribute to inclusive solutions for the long-term, (4) consider context conditions and (5) foster communication and learning.

## Introduction

Global responses to societal challenges in terms of sustainability are called for by the 2030 Agenda for Sustainable Development and the New Urban Agenda adopted at the United Nations’ HABITAT III conference (Kabisch et al. 2017). From there, the International Union for Conversation of Nature (IUCN) has shaped the term Nature-based Solutions (NBS) to highlight the importance and opportunities of “actions to protect, sustainably manage and restore natural or modified ecosystems, that address societal challenges [...] effectively and adaptively, simultaneously providing human well-being and biodiversity benefits” (Cohen-Shacham et al. 2016, p.xii). As a further response to the need for innovative solutions to address global societal challenges, the European Commission’s Research and Innovation policy on NBS supported the view on integrated innovative solutions. The European Commission defined NBS as “solutions that are inspired and supported by nature, which are cost-effective, simultaneously provide environmental, social and economic benefits and help build resilience” (European Commission 2016).

In the urban context, NBS have been regarded as an inclusive umbrella concept of established urban ecosystem-based approaches, such as ‘urban ecosystem services’, ‘green–blue infrastructure’, ‘ecological engineering’, or ‘natural capital’ (Frantzeskaki et al. 2019) all highlighting the potential of implementing nature elements in urban areas with a particular aim to mitigate and adapt to climate change (Kabisch et al. 2016; Hobbie and Grimm 2020) and other societal challenges rather than using technical solutions only (Pauleit et al. 2017; Raymond et al. 2017; Babí Almenar et al. 2021). Despite the immense number of academic publications on the topic (Bayulken et al. 2021), uptake of the NBS terminology in planning and practice has been limited and in cases fragmented to some frontrunning cities (Grace et al. 2021; Moosavi et al. 2021). A number of barriers for this uptake were identified and intensively discussed, including the critique on the concept itself and its lack of specificity in terms of how it can or does transform urban planning and governance (Baur et al. 2015; Escobedo et al. 2019; Krauze and Wagner 2019), the limited amount of qualified syntheses of NBS implementation examples and their measurable outcomes (Grace et al. 2021) as well as institutional barriers and path-dependencies of existing urban systems or lack of drivers of change (Davies and Lafortezza 2019; Dignum et al. 2020; Wamsler et al. 2020b).

In a pathway to advance the science and practice of NBS as well as to create a common understanding of NBS and their comparative benefits to conventional grey solutions, and to facilitate implementation and operationalisation, two sets of principles have been developed focusing on a landscape scale (Cohen-Shacham et al. 2019; Albert et al. 2021). IUCN (Cohen-Shacham et al. 2019) developed eight principles for successfully implementing and upscaling NBS proposing that NBS should deliver biodiversity benefits and contribute to conservation, and be evidence-based and science driven at a landscape level as overarching solutions. These eight principles illustrate IUCNs main interest in ecological conservation, and stress the importance of an integrative approach for implementing NBS at scale. In addition, Albert et al. (2021) introduced five principles to develop NBS also with regard to the landscape scale including place-specificity, evidence base, integration, equity and transdisciplinarity. To support the mainstreaming of NBS further, ICUN recently released a Global Standard for NBS based on the principles developed earlier (IUCN 2020). Following a two-year consultation process with about 770 contributions from various stakeholders (IUCN 2021), the standard more strongly balances ecological, social and economic aspects of NBS. In particular, the Global Standard promotes the eight principles for systematic deployment of NBS and the linking of NBS outcomes to local and global sustainability goals.

Although the different sets of NBS principles and the IUCN standard for NBS are well-developed, we argue that specific principles are needed with regard to an application in urban areas—with the aim to provide a spatial translation and operationalisation of the existing knowledge on participatory planning and good governance of NBS to the urban scale. Urban areas come with particular challenges for the application of the NBS approach and as such, they also require a critical reflection of ecological concepts (Tzoulas et al. 2007; Beichler et al. 2017; Conway et al. 2019). These particular challenges are outlined in the following.

First, understood as social–ecological–technical systems, cities are characterised by systems density, nestedness and interdependencies of social, ecological and technical dimensions (Frantzeskaki et al. 2021). Systems density and interrelations accelerate land use conflicts, further fuelled by global urbanisation processes. A core area of conflict unfolds between pressing commercial, residential and transport infrastructure development (with potential negative impacts on environmental quality) and safeguarding and/or developing new urban green and blue spaces for mitigating and adapting to climate change impacts ensuring a high quality of life in cities (Haaland and van den Bosch 2015; Artmann et al. 2019). This multidimensionality and interrelatedness of urban systems need to be regarded for the design and implementation of NBS which, in turn, require strategies in urban planning and governance to deal with.

Second, due to specific environmental conditions in cities, including lacking ecological connectivity, urban biodiversity differs from the regional biodiversity and species populations tend to be isolated with small habitats, exposed to disturbances and consequently being more vulnerable. Ecosystems in cities are deeply changed through anthropogenic impact resulting in altered water and soil regimes as well as novel urban ecosystems and species behaviour adapted to urban conditions (Kowarik 2011a; Alberti 2015). At the same time, urban areas are often hotspots of biodiversity and host endangered species that have difficulties to survive in modern agricultural landscapes (Ives et al. 2016). Thus, supporting urban biodiversity with NBS necessitates strategies that respond to the particular challenges and local socio-ecological conditions (Parris et al. 2018).

Third, with NBS being interventions in urban places that reconfigure values, benefits, services and uses of spaces and impact accessibility for diverse urban societies, their planning and governance is a multi-actor issue. Particularly in the context of urban sustainability transition, the planning and governance of NBS requires involvement of a diversity of stakeholder groups and the civil society in order to avoid unintended social outcomes (Dignum et al. 2020; Moosavi et al. 2021). It is the cities where participation and inclusion in decision making is asked for the simplest adaptations and interventions in public urban spaces. It is also in cities where environmental injustices may be highlighted the most—with unequal distribution of environmental threats and goods close to each other, starting already at the neighbourhood scale and with high demands for fairness in participation in environmental decision making (Baró et al. 2021).

Fourth, every city is unique and comes with its own path-dependencies related to past cultural values and planning paradigms which may continue to favour grey solutions as the dominant infrastructure option (Davies and Lafortezza 2019). Some cities were re-built over centuries, some are built only in the last decade. Some cities have faced stages of population growth followed by stages of decline and even re-growth with different land use legacies and diverse social and cultural conditions (Wolff et al. 2017). These path-dependencies still determine current urban planning and require careful strategies for sustainable transformations (Malekpour et al. 2015; Wolfram 2018).

Fifth, there may be—still—a misconception of cities as artificial landscapes separated from nature and in which transformational change in a city is regarded to be driven by technological innovations (Haase et al. 2014). In such a social–technological approach, nature and ecological innovations such as NBS do not play any significant role for sustainability transitions. Based on the previous work, we well know, however, that nature and urban life are deeply intertwined and that transformational change can happen with socio-ecological innovations (Elands et al. 2019; Dignum et al. 2020; van der Jagt et al. 2020). Pronouncing the narrative of socio-ecological innovations driven by the interconnectedness of nature and urban life would help to reconnect people with nature in cities. Reconnecting people with nature accelerates sustainability transitions through socio-ecological connections (Lin et al. 2021; Moglia et al. 2021; Oke et al. 2021), which can be framed and pushed further by NBS but requires dedicated strategies and principles.

In conclusion, integrative urban NBS principles are required that specifically address the five challenges considering i) potential development conflicts with urban systems nestedness; ii) specific urban biodiversity and related environmental conditions; iii) value plurality, multi-actor interdependencies and environmental injustices; iv) path-dependencies with cultural and planning legacies; and finally v) a potential misconception of cities as being artificial landscapes disconnected from natures.

Given the five challenges NBS strategies should consider in the urban context, the aim of this perspective paper is to complement earlier considerations about NBS principles (Cohen-Shacham et al. 2019; Albert et al. 2021 and others) by introducing five specific principles for urban NBS design, planning and implementation. With a particular view on urban systems, these principles are intended to transcend governance gaps and stimulate the discussion around urban NBS towards more effective and sustainable designs that fit the urban morphology, social, ecological and institutional dynamics.

## Five principles for urban nature-based solutions planning and governance

The rationale on how our five principles for urban NBS are formulated and discussed is based on the five specific urban challenges as outlined above. The principles further build on knowledge from multiple urban disciplines (urban ecology, urban sociology, urban design, urban planning and governance).

**Fig. 1:**
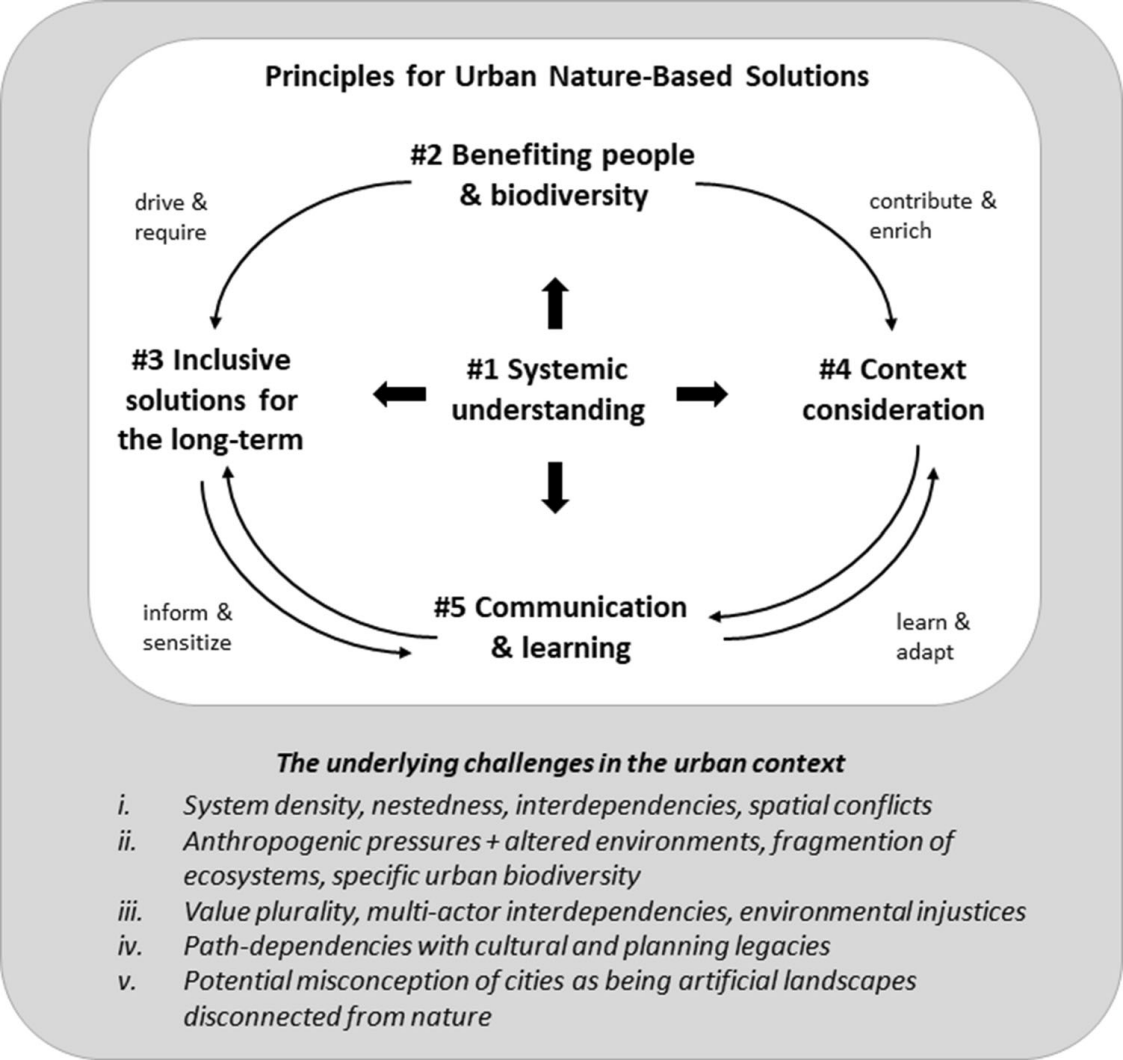
Interlinked five principles for urban nature-based solutions (NBS) illustrated with the particular underlying challenges for the application of the NBS approach in the urban context. *Note* Principle 1 “Systemic understanding” underpins all other principles. Principle 2 “Benefiting people & biodiversity” aims at a balanced delivery of multiple benefits for humans and non-humans that need to be based on understanding of the local context (Principle 4) and to be designed as inclusive long-term solutions (Principle 3) to make NBS sustainable and last over time. Principle 5 “Communication & learning” points to NBS depending on the understanding and support of citizens and is thus related to Principle 3 “Inclusive solutions for the long-term”. Principle 4 “Context consideration” and Principle 5 “Communication & learning” are similarly connected to each other in a way that NBS need to be adapted to the local context and the forms of education and communication which also depended on the local context

Our proposed five distinct principles should be regarded as a suite of guiding tenets but not in isolation. They are interlinked, show clear overlaps and integrate knowledge from academic scholarship and build on lessons learnt about planning NBS in cities (Frantzeskaki 2019; van der Jagt et al. 2020; Tzoulas et al. 2021). Figure 1 illustrates how our five principles for urban NBS are interlinked in the context of specific urban challenges.

As such, the proposed urban principles can offer a new perspective to select and screen frameworks for designing, monitoring and evaluating planning and mainstreaming for NBS, and also to ensure that they contribute to resilient and inclusive cities on the short-term and in the long-term future of cities. In essence, they respond to identifiable needs and specific urban dynamics and also provide prospects for future research. Taking an urban angle, our principles for NBS built on and extend existing principle frameworks (see Table 1 for the interlinkages). Box 1 showcases an urban application example and how the principles may translate in a real case.

**Table 1:** Proposed urban NBS principles and how they relate to practice and academic publications

Our proposed Urban NBS principles	Principles to build a common language and understanding of NBS: Relating to IUCN, Cohen-Shacham et al. (2019)	Guiding principles for a potential successful implementation of NBS: Relating to Albert et al. (2021)	Theoretical considerations for planning and implementation of NBS: Tzoulas et al. (2021)	Lessons for planning NBS in urban areas: Frantzeskaki (2019)
#1 Need for a systemic understanding: Urban NBS are integrated solutions and need to be based on a systems approach	#2: NBS can be implemented alone or in an integrated manner with other solutions to societal challenges #6: NBS are applied at a landscape scale	#5: Transdisciplinarity	#3: Transdisciplinarity #4: Polycentric governance	#6: An inclusive narrative of mission for NBS can bridge knowledges and agendas across different departments of the city and tackle with departmental disputes
#2 Benefiting people and biodiversity: Urban NBS need to ensure a balanced delivery of multiple benefits for humans and non-humans	#1: NBS embraces nature conservation norms and principles # 5: NBS maintain biological and cultural diversity and the ability of ecosystems to evolve over time	#2: Based on evidence	#1: Relational values for NBS #2: Multifunctionality of NBS	#1: NBS need to be aesthetically appealing for citizens to appreciate and protect them
#3 Inclusive solutions for the long-term: Urban NBS need to be inclusively designed, planned, implemented, and managed to appreciate long-term benefits	#4: NBS produce societal benefits in a fair and equitable way in a manner that promotes transparency and broad participation #7: NBS recognise and address the trade-offs between the production of a few immediate economic benefits for development, and future options for the production of the full range of ecosystems services #8: NBS are an integral part of the overall design of policies, and measures or actions, to address a specific challenge	#3: Integration #4: Equity	#2: Multifunctionality of NBS #1: Relational values for NBS #4: Polycentric governance	#2: Nature-based solutions create new green urban commons #5: NBS require a collaborative governance approach #3: NBS experiments require and feed into trust between the city and its citizens both for the aim of the experiment and for the experimenting process itself
#4 Context consideration: Urban NBS should respect and planned considering the local context	#3: NBS are determined by site-specific natural and cultural contexts that include traditional, local and scientific knowledge	#1 Place specificity		#7: NBS need to be designed in such a way and scale that lessons for their effectiveness can be easily harvested and as thus, to be easily replicated into other locations
#5 Communication and learning: Urban NBS should support mutual learning for sustainability transitions in cities	#7: as above	#5: Transdisciplinarity		#4: Different fora for co-creating nature-based solutions are needed that include and learn from urban social innovation

Here, we explain how our principles complement and extent the other principles and key lessons identified

## Urban NBS principle 1—Need for a systemic understanding: Urban NBS are integrated solutions and need to be based on a systems approach

### Background and challenge

Urban NBS do not exist in isolation. They are part of socio-ecological systems, as which cities can be understood (Ernstson et al. 2010), and are influenced by biophysical processes as well as social and political practices (Ernstson 2013; Moosavi et al. 2021; Tzoulas et al. 2021). Moreover, NBS are interconnected with grey urban infrastructures, the water drainage system and mobility infrastructures including streets and pedestrian paths. They can function in synergy with other urban infrastructures to (co-)create liveable, resilient, just and sustainable urban environments (Frantzeskaki et al. 2021). However, for reaching deeper leverage points in systemic transitions such as the mobility turn, they cannot only be regarded as physical infrastructure but need to be embedded in a societal process (Fischer and Riechers 2019). This interconnectedness of ecological, social and technical dimensions results in high systems complexity in which different kinds of knowledge is needed to the planning, design and management of NBS (Frantzeskaki and Kabisch 2016; Keeler et al. 2019). 

Considering systemic thinking at different spatial scales, urban planners need to ensure that core areas providing NBS such as urban forests, wetlands or large parks are protected and maintained for the whole city area, while at a neighbourhood scale street trees or other small private green patches might represent crucial NBS. Planning for NBS need to consider different spatial scales and deal with both publicly owned land and private land in order to steer collective as well as individual decisions for or against NBS (Goddard et al. 2010; Hsu et al. 2020). As such, NBS shaped by individual decisions should be considered as a ‘resource by small actions’ that contributes to NBS at a larger scale and requires specific governance approaches (Dewaelheyns et al. 2016, p. 192; van der Jagt et al. 2020).

### Implications for Urban Planning and Governance

A systems approach is fundamental to the design, planning, implementation and management of NBS. A systems approach can connect tactical with strategic urban planning, meaning that master planning guides implementation on the ground but remains open and flexible to adaptations coming from tacit (individual) knowledge, experience and learning during their implementation and environmental management (Hansen et al. 2019; van der Jagt et al. 2020). In terms of governance for NBS, a systems approach would also translate openly in the need for cross-departmental, more intersectoral collaboration. Intersectoral collaborations means getting different urban planning departments such as transport and mobility, social policy, water infrastructure, green space planning and health with their particular expertise together to plan for integrating NBS to urban fabrics in an inclusive and multifunctional way (Kabisch et al. 2016; Bush 2020; Frantzeskaki et al. 2020; Moosavi et al. 2021). NBS could act as a lens through which planners look holistically and collaboratively on the socio-ecological and technological dimensions of a city instead of planning in disconnected silos (Bush 2020; Randrup et al. 2020; Wamsler et al. 2020a). Here, NBS would support a shared view on the complex system of cities, its challenges and potential solutions and with this would help to develop a common language and building trust (Fastenrath et al. 2020). Starting such a cross-departmental collaboration through NBS projects has the potential for a long-term shift towards institutionalised sustainability transitions (Wamsler et al. 2020b).

## Urban NBS principle 2—Benefiting people and biodiversity: urban NBS need to ensure a balanced delivery of multiple benefits for humans and non-humans

### Background and challenge

Urban NBS should deliver and correspond to human needs and at the same time contribute to ecological flows and provide habitat for species diversity. In other words, urban NBS should correspond to both ‘human-oriented’ and ‘nature-oriented’ goals, which are “although not mutual exclusive, […] not always compatible” (Maller 2021, p. 3). Although knowledge on the delivery of regulating ecosystem services by NBS such as flood protection (Krauze and Wagner 2019; Kooy et al. 2020) and thermal comfort in urban areas (Kabisch et al. 2021; Ossola et al. 2021) is increasing, evidence on how NBS improve local biodiversity conditions (as also specifically required by IUCN’s NBS definition) in cities is scarce and ecological cycles are usually strongly altered or even disrupted (Parris et al. 2018). Consequently, ecological concepts and conservation goals only partly translate to urban areas (Kowarik 2011b; Kowarik and von der Lippe 2018). In addition, most urban NBS require a certain level of maintenance to preserve a state that is in line with the multiple demands that need to be met in urban areas, ranging from aesthetic appeal and public safety, to preventing health or biodiversity trade-offs or avoiding property and hard infrastructure damage (von Döhren and Haase 2015; Roman et al. 2021). Overall, planning, maintenance and monitoring of NBS needs to be responsive and flexible because “nature does not provide solutions by traditional linear, analytical means (…)” (Moosavi et al. 2021, p. 10).

Biodiversity net gain also relates to the question of connectivity within and across urban boundaries and if species are able to move through the urban matrix. Connectivity for animal species is usually concerned with connecting similar habitat types and considering the needs of target species (Parris et al. 2018; Ersoy et al. 2019). Under the concept of green infrastructure, networks of green spaces are supposed to be multifunctional supporting wildlife mobility and human mobility, air or water flows (Hansen and Pauleit 2014). To which degree this multifunctionality can be created in urban areas, still needs to be investigated. For example, restoration of urban river corridors needs to integrate social and ecological considerations and balance between recreational use and biodiversity protection (Zingraff‐Hamed et al. 2017).

### Implications for urban planning and governance

Despite potential conflicts and the need for compromise, NBS can be designed as novel or near-nature ecosystems that provide habitat functions for a diversity of species (Apfelbeck et al. 2020). Regarding the use of native versus non-native plants, Berthon et al. (2021, p. 6) note that “planting native species serves as a useful rule to increase *overall* biodiversity in urban green spaces” but at the same time also point to the fact that non-native may provide equally or similarly valuable functional traits. Urban planting strategies will usually require a mix of natives, and non-natives that are better adjusted to urban environmental conditions. In case these plants provide resources for other species they can be considered a contribution to biodiversity conservation or even a net gain (Berthon et al. 2021).

While the ideal of NBS as functioning ecosystems need to be reconsidered for urban areas, NBS can and should be inspired by local natural ecosystems and designed as spaces that require little maintenance, are relatively stable in the long-term and fulfil both human- and nature-oriented purposes, i.e. prairie gardens in the US or xeric gardens in arid regions (Ignatieva et al. 2020).

Overall, NBS features that are addressing ecological benefits and those that promote ecosystem service provisioning, usability and aesthetic appeal should both be considered while fostering synergies and avoiding trade-offs (Hansen et al. 2019). As these issues are complex, they require careful interdisciplinary knowledge from ecology and landscape design and even environmental psychology. The importance of the principle lays in the acceptance of the intrinsic value of nature for biodiversity and for us as humans equally. We are here with Maller (2021, p. 2) who discussed how NBS could be used as a lens through which urban areas could be designed or governed as “…places where multiple species and ecosystems are encouraged to flourish, including, but not limited to, humans.”

## Urban NBS principle 3—Inclusive solutions for the long-term: urban NBS need to be inclusively designed, planned, implemented and managed to appreciate long-term benefits

### Background and challenge

Decisions for urban NBS implementation requires local knowledge and integration of cultural context in terms of inclusive design considering all dimensions of socio-environmental justice (distributive, procedural, interactional or recognition; see Kabisch and Haase 2014; Baró et al. 2021; Pineda-Pinto et al. 2021). This inclusivity in design of NBS is particularly relevant in the urban context where a high population density, diverse citizen’s demands and vulnerabilities are compressed. Tzoulas et al. (2021, p. 339) argue that “the need to integrate cooperative, competing and conflicting interests in the implementation of [NBS] necessitates polycentric governance”; meaning a diversity of arrangements “that allow multiple, overlapping, semi-autonomous decision-makers to cooperate, compete and resolve conflicts between each other” (p.338, referring to Carlisle and Gruby 2019). The cultural benefits and contributions of nature need to be understood which in turn requires an integrated and inclusive approach to design and planning in terms of co-creation and co-design of NBS responding to the needs of citizens (Mattijssen et al. 2017; Buijs et al. 2019; van der Jagt et al. 2019; Frantzeskaki et al. 2021) while contributing to procedural and interactional justice.

NBS need be to planned in a way to have equitable distribution of benefits throughout their lifecycle (Zuniga-Teran et al. 2020). In this regard, the long-term effectiveness or performance of NBS and its potential outcomes—be it synergies or eventually trade-offs—need to be considered and discussed openly and transparently (Gómez Martín et al. 2020). This includes interdisciplinary approaches for analysing potential long-term outcomes in addressing the initial challenge, the multiple additional benefits or trade-offs (European Commission 2021; Grace et al. 2021; Maller 2021). For instance, if maintenance costs are not considered, instalments of sustainable urban drainage systems might lose their performance as well as aesthetic appeal. NBS in housing areas may involve unexpected upgrading of neighbourhoods and related prices in the long-term (Shokry et al. 2020; Wamsler et al. 2020a). A rise in property values or rental prices close to a NBS site can eventually worsen the situation for socially disadvantaged groups for which the NBS was initially aimed for providing a public benefit (Tozer et al. 2020). This needs to be further examined in the context of different gentrification models and processes not in spite of them with a sole focus on NBS implementation in place.

### Implications for urban planning and governance

Due to the potential trade-offs, it should not be taken granted that all citizens welcome NBS in their direct surroundings and that they might have individual interests in conflict with providing space for NBS. Balancing these effects means an inclusive NBS governance in which a diverse set of development options are discussed openly and transparently with a range of stakeholders (see Seddon et al. 2020 for inflexible forms of governance acting as barriers to uptake NBS and Fors et al. 2021 on participation during management of green space). Potential risks need to be communicated transparently throughout NBS design and implementation processes in order to find strategies to prevent or potentially overcome them.

An inclusive and thus, just approach to planning NBS will entail a holistic assessment and understanding of multiple benefits, potential trade-offs as well as an understanding of how their multifunctionality can be secured or hampered due to socio-economic and cultural contextual aspects throughout their lifecycle (Cousins 2021; Giachino et al. 2021; Pineda-Pinto et al. 2021). Transdisciplinary approaches and wide participation may increase the acceptance of NBS and translate in innovative instruments that can strategically prevent trade-offs to appear on the long-term (Moosavi et al. 2021).

## Urban NBS principle 4—Context consideration: urban NBS should respect and be planned considering the local context

### Background and challenge

In our understanding, urban NBS are an alternative type of infrastructure that helps adapting and mitigating societal challenges in a specific local, cultural and ecological context. Fully understanding the context of cities is challenging given the presence and density of manifold socio-ecological structures, different cultural and population age groups and with this different demands and values. Other cases can inspire but NBS should not be simply imported und replicated in a technical manner. Design, implementation and maintenance should consider potential adaptations related to the local social–cultural, ecological and economical context (Fastenrath et al. 2020).

Local and context-specific challenges may include to prioritise NBS implementations to areas with intensified urban heat island, areas with lower shares of green spaces and thus, lower environmental quality while aiming for improving health and well-being of residents (Kabisch et al. 2020; Kabisch and Kraemer 2020). Considering the socio-ecological context dynamics, NBS need to be designed to fit the context and produce (co-)benefits that restore local ecological flows and enrich biodiversity and the local community (Gomez Martin et al. 2020). For example, Grace et al. (2021) identify a knowledge need for NBS adapted to water-scarce environments, pointing to the requirement to match NBS to the biophysical context (Meerow et al. 2021).

### Implications for urban planning and governance

NBS should be socio-culturally embedded in cities, given that they will be ‘local interventions’ that will alter or disrupt local meanings of place, transform senses of place and connections with urban nature of local communities (Breen et al. 2020). NBS need to encounter and be designed in such a way to ‘enrich’ if not fit in the cultural context of existing urban nature (Nagendra and Mundoli 2019; Basu and Nagendra 2020) which in turn not only benefits health and well-being but has a dedicated meaning to people. The planning of NBS in urban areas needs to further consider this potential meaning to people also through the consideration of the history of places and path-dependencies in urban planning, industrial or financial systems (van der Jagt et al. 2020). Understanding the potential and opportunities for NBS integration requires considering potential enabling but also hindering factors that may be rooted in historical decisions (Davies and Lafortezza 2019). Zwierzchowska et al. (2021) showcase how history of housing design and planning in the cities of Poznan, Poland and Berlin, Germany informed and can inform different positioning opportunities for urban parks and for other types of NBS. They also evidenced the historical trajectory for green spaces as urban commons in cities that is an enabling socio-cultural context for the development of new NBS as part of urban green commons.

## Urban NBS principle 5—Communication and learning: urban NBS should support mutual learning for sustainability transitions in cities

### Background and challenge

NBS in urban environments have the potential to (re)establish connections of people with nature and in this way may also contribute to pro-environmental behaviour and increased awareness of the significance of sustainability in everyday urban life (Soga and Gaston 2020; West et al. 2020). Engaging in pro-environmental behaviour through environmental stewardship practices may also be related to human well-being, social cohesion and happiness which in turn may lead to increased nature interaction and positive conservation attitudes (Buijs and Jacobs 2021). As Randrup et al. (2020) name it “nature-based thinking” via nature-based learning is a pathway for sustainable urban development. The advantage of relating to NBS as a concept may here lay in its simplicity to highlight the importance of nature and thus, helping to mainstream planning, taking care for and living with nature or NBS as the ‘new normal’ (Cohen-Shacham et al. 2019; Davies and Lafortezza 2019; Moosavi et al. 2021).

Research in selected European cities has shown that near-natural greening is considered as attractive for humans and may provide similar opportunities for recreational activities compared to a conventional park or square (Fischer et al. 2018). However, there might be a need to transport the message that NBS have purpose even if they might not have the traditional aesthetic appeal of ornamental green by providing ‘cues for care’ by means of design that signal intentionality and human presence in such spaces (Li and Nassauer 2020). An increased sense of belonging and attachment might be related with aesthetical appeal of a NBS and support appreciation, recognition and awareness of the benefits of nature in cities (Frantzeskaki 2019; Gómez Martín et al. 2020; Bayulken et al., 2021).

### Implications for urban planning and governance

In terms of making urban NBS part of sustainability transitions, they should be part of transdisciplinary and citizen-based environmental learning as well as awareness strategies and campaigns. Creating knowledge and increasing awareness about the benefits of nature in cities as a response to pressing global challenges, not only with the general public but also local decision-makers, may help creating an argument in local planning budget deliberations and increases support of implementing NBS compared to pure technical solutions (Davies and Lafortezza 2019) and would also help maintaining nature in cities. Citizen involvement in sustainability transitions involves among others, urban experimentation such as in living labs (Dignum et al. 2020), city-to-city learning, joint citizen walks and excursions, workshops all aiming at raising awareness and starting citizen dialogues (Frantzeskaki et al. 2020) which may help to avoid contestation (Wamsler et al. 2020b).

Planning and implementation of NBS should be informed by citizens’ concerns and preferences to support positive relations with urban nature. In this context, learning should be mutual, also aiming at expanding awareness for citizens’ needs. In addition to this, NBS efforts should be culturally sensitive and allow citizens to express their ideas of aesthetic appeal and functioning, balancing between subjective values, preferences, uses, conflicts and beliefs on the one side and goals of NBS planners for services delivery on the other side (Beumer and Martens 2015).

**Figure:**
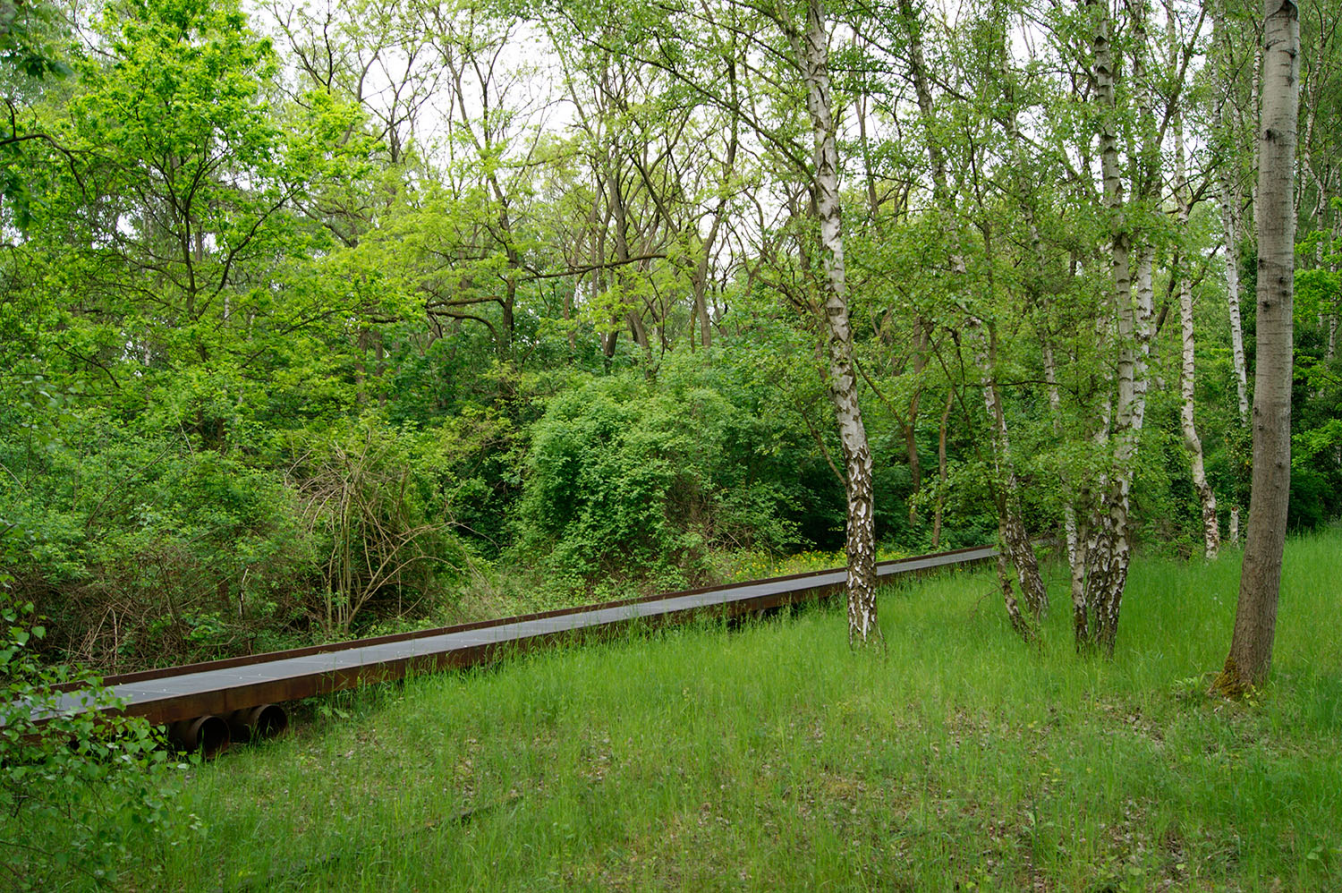
BOX 1 An urban application example of the five principles for urban nature-based solutions - Schöneberger Südgelände, Berlin

**Table:** 

**Schöneberger Südgelände, Berlin, Germany** is an iconic example of how urban biodiversity can be maintained and promoted. The former wasteland was transformed into a nature park that maintains the appeal of an urban wilderness.	
**#1 Need for a systemic understanding:** The area was developed as part of a larger green corridor through the city and provides ecological and social–cultural benefits in a synergistic manner.	
**#2 Benefiting people and biodiversity:** The area is designed to ensure both recreational use by humans and biodiversity protection. Visitors are guided on elevated paths through sensitive areas that provide a physical access barrier but allow nature experiences.	
**#3 Inclusive solutions for the long-term:** Maintenance ensures both, further evolvement as urban wilderness as well as preservations of valuable habitats. The dry grassland is grazed with sheep to persevere this species-rich succession stage. In wooded parts, succession happens (as far as public safety allows).	
**#4 Context consideration:** When the wasteland was supposed to be redeveloped, a citizen initiative protested due to the outstanding ecological value of the area, resulting in the conservation and protection of the area and nowadays allows citizens to experience a specific kind of urban nature.	
**#5 Communication and learning:** The park aims to promote nature experience. Design with elevated paths, art works and the presentation of remnants of the prior use as a railway station contribute to a unique character that presents the urban wilderness as valuable and intentional. In addition, educational information about the ecological value is provided.	
For more information see:	
https://gruen-berlin.de/en/projects/parks/natur-park-schoeneberger-suedgelaende/about-the-park	
https://www.berlin.de/senuvk/umwelt/stadtgruen/gruenanlagen/de/gruenanlagen_plaetze/schoeneberg/naturpark_suedgelaende/index.shtml	
Lachmund (2013)	

## Concluding remarks

Hundreds of scientific papers are now out all using and referring to the term, concept or framework of NBS. In 2016, we were among the first discussing perspectives on indicators, knowledge gaps, barriers and opportunities for action for NBS (Kabisch et al. 2016) and also compared NBS with other recently evolved concepts (Pauleit et al. 2017). What we have seen since from the many papers published in scientific journals, special issues, or synthesis reviews is, that all they have in common is some request to make the term “NBS” operational.

Acknowledging existing principles being important for a broader landscape scale, with this perspectives paper, we aim to stimulate discussions on the planning and governance of NBS in urban areas. We introduced five principles for urban NBS because cities come with specific challenges. We conceptualised our principles against five major challenges and used recent research and urban applications for illustration. Our five principles for urban NBS offer an extended, interlinked perspective when NBS are to be implemented in urban areas but may also guide future research on urban NBS and sustainability transitions.

With the consideration of our five principles for urban NBS, we hope to contribute to an increased appreciation of nature in cities in which the beneficial contribution of nature is considered, given a proper weight in collaborative decisions and becomes a core solution, while still considering that NBS build on living organisms and ecological processes that require context-adaptation, specific conditions, appreciation of citizens and long-term care.

For some principles, we mentioned potential trade-offs related to NBS. More discussion is needed in the context of emerging contestations and conflicts that may arise when NBS are being implemented, even with best intentions. Future research may help to assess whether it is possible to avoid or partially or fully prevent trade-offs, and if not, how schemes to prioritise and decide between different actors, functions and beneficiaries could look like.

With the Nature Editorial (2017), now nearly five years later, we are very optimistic that we will be reaching the point soon, when the word ‘nature-based solution’ is as broadly and axiomatically used in science, policy and society as we already use the words ‘sustainable development’ or ‘biodiversity’.
